# Bio-Based Adhesives and Evaluation for Wood Composites Application

**DOI:** 10.3390/polym9020070

**Published:** 2017-02-17

**Authors:** Fatemeh Ferdosian, Zihe Pan, Guchuhan Gao, Boxin Zhao

**Affiliations:** Department of Chemical Engineering, University of Waterloo, Waterloo, ON N2L 3G1, Canada; fferdosi@uwaterloo.ca (F.F.); z26pan@uwaterloo.ca (Z.P.); g22gao@uwaterloo.ca (G.G.)

**Keywords:** bio-based adhesives, lignin, starch, protein plants, adhesive evaluation, wood composites

## Abstract

There has been a rapid growth in research and innovation of bio-based adhesives in the engineered wood product industry. This article reviews the recent research published over the last few decades on the synthesis of bio-adhesives derived from such renewable resources as lignin, starch, and plant proteins. The chemical structure of these biopolymers is described and discussed to highlight the active functional groups that are used in the synthesis of bio-adhesives. The potentials and drawbacks of each biomass are then discussed in detail; some methods have been suggested to modify their chemical structures and to improve their properties including water resistance and bonding strength for their ultimate application as wood adhesives. Moreover, this article includes discussion of techniques commonly used for evaluating the petroleum-based wood adhesives in terms of mechanical properties and penetration behavior, which are expected to be more widely applied to bio-based wood adhesives to better evaluate their prospect for wood composites application.

## 1. Introduction

Conventional adhesives for wood composites are based on four major synthetic thermosetting resins: phenol formaldehyde (PF, alkaline catalyst salt), urea formaldehyde (UF, acidic catalyst salt), melamine-formaldehyde (MF), and polymeric diphenylmethane diisocyanate (pMDI) resin [1,2]. PF resins are generally synthesized from the reaction of phenol with formaldehyde under alkaline condition [3]; they can provide great adhesive strength, high mechanical strength [4], excellent thermal stability [5], low initial viscosity and great moisture resistance [3,6,7]. They are widely consumed in the manufacture of products such as oriented strand board (OSB), softwood plywood, and siding that requires exterior exposure durability [2]. UF resins are mainly used in manufacturing of particleboard and medium density fiberboard (MDF) where the dimensional uniformity and surface smoothness are the main requirements [8]. UF resins can be formulated for a wide curing temperature ranging from room temperature to 150 °C for a moderate curing time. In comparison to PF resins, UF resins require shorter pressing time and lower pressing temperature, resulting in a faster production rate and lower energy consumption; they are appropriate for making decorative products due to a light color [2]. MF resins are typically used for various applications such as paper treating, decorative laminates, and paper coating. They are usually blended with UF resins for specific applications due to their relatively high price [2]. pMDI have the characteristics of fast curing, formaldehyde-free emission, small loading quantity, and good weather resistance; they are used primarily in the manufacture of OSB [2,9]. Formaldehyde has been categorized as a carcinogenic and toxic material, with acute oral toxicity (LD50) of 100 mg/kg (Rat) based on the material safety data sheet (MSDS) of science lab. However, pMDI is much less toxic than formaldehyde, with acute oral toxicity (LD50) of >2000 mg/kg (Rat) based on the MSDS of Demilec (Dallas, TX, USA) LLC. The production of these resins has relied on non-renewable petroleum resources. Therefore, there has been a growing interest in the development of environmentally friendly wood adhesives from renewable resources.

Various biomass resources such as lignin [10,11,12], starch [13,14], plant proteins [12,15,16,17], tannin [18], bark [19,20], and vegetable oils [21] have been used as a renewable feedstock to synthesize bio-based adhesives. The main focus of this review paper is on the application of bio-adhesives for engineered wood panels. We have reviewed the recent literature on the development of bio-based wood adhesives based on three major types of biopolymers: lignin, starch, and plant proteins.

## 2. Lignin

### 2.1. Chemistry of Lignin

**Figure 1:**
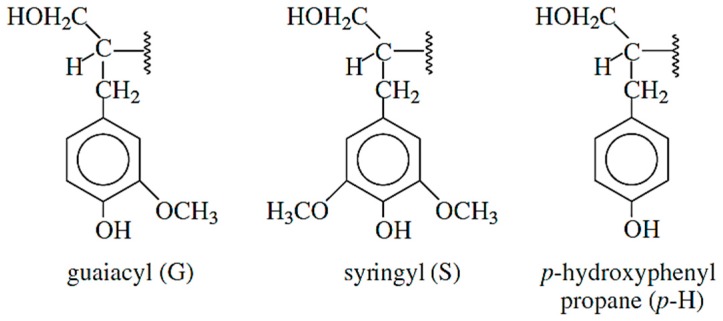
The three structural units of lignin [25], reproduced with permission from Elsevier.

Lignin has a phenolic structure with a potential to be used as a replacement of phenol in the synthesis of phenolic resins to formulate wood adhesives. In addition, lignin has several properties which are attractive to be used as adhesives, including high hydrophobicity, low glass transition temperature (*T*_g_) (ranging from 60–90 °C for organosolv lignin [22] and 100–170 °C for lignin obtained from most thermomechanical pulping [23]), and low polydispersity [24]. Lignin is made of three monomers: coniferyl alcohol, synapyl alcohol, and *p*-coumaryl alcohol, which are linked together mainly by ether linkages and condensed linkages. The final structure of lignin is composed of three units, namely, guauacyl (G-type), synringyl (S-type), and *p*-hydroxylphenol propane (*p*-H-type) units, as illustrated in Figure 1 [25]. The relative content of these units in the structure of lignin depends on the original source of lignin (softwood and hardwood) and the delignification process [25,26,27].

Lignin-based adhesives can be divided into two groups: phenol-formaldehyde adhesives and formaldehyde-free adhesives. In the former group, lignin is used as a partial replacement of phenol, while in the latter lignin reacts with chemicals other than formaldehyde to generate bio-adhesives with less toxicity.

### 2.2. Lignin-Based Phenol-Formaldehyde Adhesives

For synthesis of lignin-based phenol-formaldehyde (PF) resin, formaldehyde could be linked to the free ortho positions of the phenolic groups of lignin via electrophilic substitution [28]. Based on the chemical structure of lignin, both ortho positions (C3 and C5) of the S-type unit are occupied by methylene groups and cannot react with formaldehyde. H-unit and G-unit have two and one free ortho positions, respectively, on the aromatic ring to react with formaldehyde; they are more reactive than S-unit as a replacement of phenol in the production of PF resins [25]. It has been revealed that the softwood lignin is mainly composed of G unit in comparison with hardwood lignin and agricultural residues, making it more desirable for the synthesis of PF resins [26,29]. Hence, it is required to know the exact chemical structure of lignin prior to the synthesis of lignin-based phenol formaldehyde resin. Tejado et al. [25] characterized the physico-chemical properties of various types of lignin including Kraft pine lignin, soda–anthraquinone flax lignin, and ethanol–water wild tamarind lignin to determine which one is more suitable for the synthesis of PF resins. It was concluded that Kraft lignin is the best candidate for the substitution of phenol because it has more free-ring positions (mainly made of G units) with a higher *M*_w_ and a greater thermal decomposition temperature than the other types of lignin.

Several studies have been performed to evaluate the potential utilization of different types of lignin including lignosulfonates, Kraft lignin, organosolv lignin, enzymatic hydrolysis lignin, and soda lignin in the synthesis of phenol-formaldehyde (PF) resin for wood adhesives [30,31,32,33]. The incorporation of unmodified lignin to phenolic resin formulation is limited to a low replacement level and shows a reduction in adhesion strength with a longer pressing time [34] because lignin has lower reactivity toward formaldehyde compared to phenol. Hence, lignin requires either chemical modification or molecular fractionation to improve its reactivity in the production of PF resins. For this, various chemical modifications have been developed such as demethylation [35], methylolation [24,27,36,37], phenolation [30,38,39,40], and sulphonation [38,41].

Akhtar et al. [42] used lignosulfonate (LS) as a replacement of phenol in the synthesis of PF resin at a relatively high replacement level (up to 50 wt %). PF resin composed of 50% of LS had better strength properties in comparison to the commercial adhesives with a great economic effect on the formation of PF resin. Twenty percent substitution of phenol by LS resulted in a bio-adhesive with the highest values of shear strength in both wet and dry conditions. Domínguez et al. [43] investigated the chemical structure, thermal stability, and rheological behavior of a lignin-based PF resin containing a 30 wt % of methylolated softwood ammonium lignosulfonate. The results showed that the chemical structures of a typical PF resin and the modified lignin-based PF resin were similar without any significant difference. However, the addition of lignin into the formulated PF resin improved the thermal properties and varied the flow behavior of PF resin from Newtonian to Pseudoplastic type. Rheological behavior of both the modified and unmodified PF systems was predicted by various models including the Maxwell, Ellis, and Cross models. El Mansori et al. [44] optimized the proportion of formaldehyde in hydroxymethylation of calcium lignosulfonate to obtain a resin with the best performance for exterior-grade wood particleboard applications. It was found that the optimum level of hydroxymethylation depended on the molar mass of lignin. Lignin with a lower molecular mass led to a higher hydoxymethylation level and showed a better modulus of elasticity (MOE). The curing behavior and the curing kinetics of a PF resin mixed with ammonium salt lignosulfonate (ALS) filler or methylolated ALS (MALS) filler was studied, showing that the curing rate decreased with increasing content of filler [45]. It was also found that the Williams-Landel-Ferry (WLF) model could provide a better description for viscosity as a function of conversion in comparison to the dual Arrhenius model, particularly above the gelling point.

Matsushita et al. [28] investigated the influence of Kraft lignin on the surface characteristics of PF resin at two substitution levels of 7 wt % and 15 wt % using the contact angle measurements and inverse gas chromatography (IGC). It showed that the acid-base component of the work of adhesion between resin and water decreased, whereas the Lifshitz-van der Waals component increased in the presence of lignin. It means that lignin-based PF resins showed better water resistance compared to the conventional PF resins. In another study, a Kraft lignin-based PF resin (KLPF, 50 wt %) was produced and used as the binding agent for an oriented strand board [46]. The modulus of elasticity was improved from 2400 N/mm^2^ to 2539 N/mm^2^ by introducing 50 wt % Kraft lignin into PF resin. Other parameters including modulus of rapture, internal bonding strength, water absorption, and swelling thickness remained approximately constant in the presence of Kraft lignin. It suggested that KLPF resin could be used as a promising binder in the manufacture of oriented strand board panels. Siddiqui et al. [47] conducted a study on the production of PF resins derived from the de-polymerized softwood Kraft lignin. The synthesis process was optimized using Box-Behnken Design (BBD) based on the three variables: of the substitution levels of phenol by lignin, the average molecular weights of lignin, and the molar ratios of formaldehyde to phenol, aiming to decrease the curing temperature and to improve the bonding strength of the synthesized lignin-based PF resins. Kouisni et al. [48] recovered lignin from Kraft black liquor using a pilot press filter or used a pilot belt filter as a raw material for the synthesis of phenol-formaldehyde resins. It was demonstrated that both types of the recovered lignin had similar physico-chemical properties with high purity. However, there were some differences in the solid content of the collected lignin and the operation speed of these two separation methods. The purified lignin was then incorporated in the synthesis of PF resin up to 30 wt % of the phenol without compromising the plywood shear strength.

Enzymatic hydrolysis lignin (EHL) has also been used in the production of PF resin. Jin et al. [5] investigated the influences of the ratio of EHL to phenol and the NaOH content on the properties of plywood bio-adhesives. The prepared bio-adhesives containing 5–20 wt % of EHL and 2.5 wt % of NaOH met the minimum requirement for the first grade plywood based on the Chinese National Standard GB/T14732-2006, while the measured wet bonding strength is much higher than the standard value for first grade plywood. In a similar study, Qiao et al. [38] reported that 30 wt % of EHL would be the optimum content for first grade plywood. The adhesion strength of 60 wt % of lignin substitution was in an accepted range, but the thermal properties deteriorated at this high content of lignin.

Park et al. [49] prepared a lignin-based PF resin for coatings and composites with a particular focus on the way to purify lignin to form a product with uniform thermal stability. An extracted bagasse lignin was subjected to cyclohexane/ethanol extraction in a soxhlet to remove such impurities as waxes, lipids, tannin, and low molecular weight lignin. The purified lignin underwent the hydroxymethylation process to become more reactive for the synthesis of PF resin. The content of lignin varied from 10–40 wt % in the final products. The resin containing 30 wt % lignin exhibited the best water resistance property. In another work, a phenolic resin was prepared from partial replacing of phenol with a methylolated organosolv lignin for bonding particleboards [37]. When the lignin substitution reached 30 wt %, the physical and water absorption properties of the prepared particleboards were comparable with those bonded with a control PF resin. Cetin et al. [50] characterized the performance of the particleboards bonded by phenolated-organosolv lignin-formaldehyde resin in comparison with a commercial and an unmodified lignin-based PF resin. It was found the phenolated lignin exhibited better mechanical properties than the unmodified lignin. When the content of lignin is in the range of 20–30 wt %, the internal bonding (IB) strength in wet and dry tests is comparable with those of the commercial PF particleboards. Vazquez et al. [51] optimized the copolymerization of phenol, formaldehyde, and methylolated acetosolv pine lignin for manufacturing plywood boards. An experimental protocol was designed based on a 2 × 2 × 2 factorial design with three variables including the phenol: lignin ratio (P/L), the formaldehyde: phenol ratio (F/P), and the soda: phenol ratio (S/P). The gel point was found to increase linearly with increasing the soda percentage (S%) and decrease with increasing S/P ratio by 1.75 times. The free phenol content showed a complex dependency on all three variables. The knife test of pine boards was noticeably affected by S% and S/P ratio, whereas these variables had no effect on the knife-test results of the eucalyptus boards.

### 2.3. Formaldehyde-Free Lignin-Based Adhesives

Formaldehyde has been categorized as a carcinogenic and toxic material, with LD_50_ of 65 mg/kg, by the Environmental Protection Agency (EPA) in 2008 [52]. Therefore, many efforts have been made to remove formaldehyde from wood adhesive formulation and to develop environmentally friendly adhesives. Most published works and industrial trials have used lignin to partially replace phenol-formaldehyde and urea-formaldehyde resin. There are only a few studies on synthesis of lignin-based adhesives without formaldehyde. For instance, El Mansouri et al. [53] developed a novel process to substitute formaldehyde with glyoxal, a non-volatile non-toxic aldehyde but less reactive than formaldehyde. In this process, calcium lignosulphonate with molecular weight (*M*_w_) of 4634 g/mol was reacted with glyoxal under an alkaline condition. The modified lignin was mixed with pMDI and applied as wood adhesives for particleboard application. The prepared formulation met the requirement of international standard specification (EN 312) for exterior-grade panels and showed comparable reactivity to formaldehyde-based adhesives. In another work, El Mansouri et al. [44] investigated the performance of the glyoxalated lignin (GL) in mixture of pMDI in the production of particleboard. It was demonstrated that the addition of triacetin enhanced the formation of methylene bridges between lignin aromatic nuclei. The formulation containing 60/40 wt % of GL/pMDI adhesive (with a loading content of 8% on dry wood) showed excellent internal bonding (IB) strengths in dry and boiled conditions for exterior-grade boards. This technology has been applied on two different types of low-molecular-mass lignin to completely eliminate formaldehyde from wood adhesive formulation [54]. The mixture of glyoxalated lignin, mimosa tannin and pMDI, up to 80 wt % natural polymers, yielded a good internal bonding and met the requirements for interior-grade panels. In addition, it has been found that lignin with a lower molecular weight in the formulated pMDI showed a better internal bonding strength. Moreover, the introduction of triacetin into the lignin-based adhesives enhanced the curing process of the methylolated lignin (ML) with pMDI and improved the modulus of elasticity (MOE) value. The ML/pMDI formulation containing around 30% pMDI showed good internal bonding strength and met the standard requirements. Navarrete et al. [55,56] prepared a novel bio-based adhesive derived from a low molecular mass lignin and tannin without incorporating any synthetic resin. The low molecular mass lignin is a by-product of formic acid/acetic acid pulping of wheat straw. Firstly, lignin was modified with glyoxal under alkaline condition, then mixed equally with tannin, followed by crosslinking with hexamine (5 wt % of tannin). The results showed that the internal bond strength of the bonded panel met the requirement of interior panel standard (EN 312). In addition, the lignin/tannin/hexamine binder classified as an effective zero formaldehyde emission based on the desiccator method (JIS A5908, in which the specimen (150 mm × 50 mm × 4.5 mm) is placed in a 10 L desiccator for 24 h. The formaldehyde released from the bonded panel is absorbed by 300 mL distilled water in a petri dish. The absorbed formaldehyde is then determined photometrically at 412 nm in a UV spectrophotometer).

**Figure 2:**
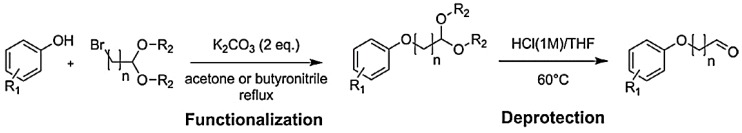
Formation of aliphatic aldehyde groups onto phenolic compounds (*n* = 1 or 2, R_1_ and R_2_: CHO, OMe or aliphatic chain substituents) [57], Reproduced with permission from Elsevier.

Foyer et al. [57] designed an innovative procedure to synthesize formaldehyde-free resol via a two-step modification process. In this process, an acetal group was grafted on a phenolic group of lignin; the formed acetal group was then de-protected into an aliphatic aldehyde group through the acid-catalyzed hydrolysis process (as illustrated in Figure 2). Afterwards, the obtained lignin-based aldehyde compounds including 4-hydroxybenzaldehyde, vanillin, and syringaldehyde reacted with phenol under alkaline condition to synthesize resol. The prepared resol resins showed good thermal stability and char yield. In addition, the reactivity of the aldehyde precursor was studied with respect to the nature and position of aromatic substitution prior to the aldehyde function [58]. It was found that the original bio-based compounds were not sufficiently reactive for the production of resol; the functionalization process could enhance their reactivity toward phenol.

Geng et al. [59] prepared a formaldehyde-free wood adhesive using Kraft lignin and polyethylenimine (PEI) for the manufacture of plywood. The optimum curing condition for this bio-adhesive in a hot press was 9 min at 140 °C. The sample containing lignin/PEI with a weight ratio of 2:1 showed the highest shear strengths (including dry shear strength, water-soaking-and drying strength, boiling water test/dry strength, and boiling water test/wet strength) and the greatest water resistance. It was found that the premixing time (>40 min) and the molecular weight (*M*_w_) of PEI ranging from 70,000 to 150,000 had negligible effects on plywood shear strength. In a similar work, a mixture of demethylated Kraft lignin (DKL) and polyethylenimine (PEI) was examined by lap-shear test [60]. The FTIR studies of the curing characteristics showed that the phenolic groups of lignin were firstly oxidized to generate quinones and then reacted with PEI. The influence of the pre-mixing time (10–120 min), curing conditions (press time from 3–7 min, press temperature from 100–160 °C), solid content (14–25 wt %), DKL/PEI weight ratio (1:4 to 4:1), and the molecular weight of PEI (10,000–750,000) on the shear strength of the formulated adhesives were also investigated. It was found that the pre-mixing time and the molecular weight of PEI had insignificant effects on the shear strength. The highest shear strength was obtained when the adhesive was formulated with 1:1 DKL/PEI and cured at 120 °C for 5 min. Yuan et al. [61] studied the physicomechanical properties of a modified ammonium lignosulfonate/PEI mixture as a green binder in preparation of medium density fiberboard (MDF), where ammonium lignosulfonate (AL) was oxidized in the presence of H_2_O_2_ under alkaline condition and then mixed with PEI. The optimum performance for furniture grade MDF was obtained at 20 wt % binder with AL/PEI weight ratio of 7:1 after curing at 170 °C for 7 min. The XRD results indicated that the crystalline structure of wood composites remained unchanged in the presence of either modified AL or unmodified AL, while the relative crystallinity of the composites was significantly improved. William et al. [34] incorporated lignin derivatives including hydroxypropyl and hydroxyethyl lignin in formulation of wood adhesives with polymeric methylene diphenyldiisocyanate (PMDI) and hexamethoxy-methyl melamine (HMMM). The presence of 50–60 wt % of lignin derivatives was required in the formulated melamine resin to show the acceptance results. However, when the lignin content reached 60 wt %, the adhesion strength of the prepared pMDI reduced by approximately 20%.

An innovative thermoset resin was synthesized from the combination of polyfurfuryl alcohol (PFA) and lignin at two different lignin contents of 20 and 30 wt % [62]. In the lignin-furfural resin system, furfural and lignin were used to replace formaldehyde and phenol in PF resins, respectively. The SEM result showed that the prepared matrix is monophasic; one relaxation peak was detected by DMA. The introduction of lignin increased the glass transition temperature of pure PFA resin from 78–87 and 101 °C for the combined matrixes containing 20 and 30 wt % of lignin, respectively, suggesting the presence of lignin led to the formation of a slightly stiffer matrix with better impact strengths. Similarly, Dongre et al. [52] worked on lignin-furfural based adhesives. In this study, lignin was extracted from sugar maple before and after acid hydrolysis and reacted with furfural as a crosslink agent. The effects of various factors including furfural content, pH, curing temperature and pressure on the mechanical properties were studied. In general, the hydrolyzed lignin-based adhesives showed a better performance because the lower molecular weight of lignin has better mobility and exhibited better mechanical properties. Furthermore, the adhesive was formulated at pH 0.65 without furfural (0%) and cured at 180 °C under 1.9 MPa demonstrating the best performance, comparable with PF resin (90% of PF strength). The adhesive composed of 16% furfural also showed good properties at pH 1 whereas furfural was not required to obtain acceptable mechanical properties at pH below 1.

Zhou et al. [63] characterized the effect of oxygen plasma processing time and power on the treatment of enzymatic hydrolysis lignin (EHL). The plasma process improved the physical and mechanical properties of EHL; the modulus of elasticity (MOE), modulus of rupture (MOR), and internal bonding strength (IB) increased and thickness swelling (TS) decreased with increasing the time and power of the plasma processing. Plasma treatment at 200 W for 5 min was found to be the optimal process conditions to prepare high-performance lignocellulosic composite. The treated EHL contained a high concentration of oxygen-containing functional groups (such as hydroxyl, carbonyl, and carboxyl groups), improving its bonding strength with popular fibers.

## 3. Starch

### 3.1. Chemistry of Starch

**Figure 3:**
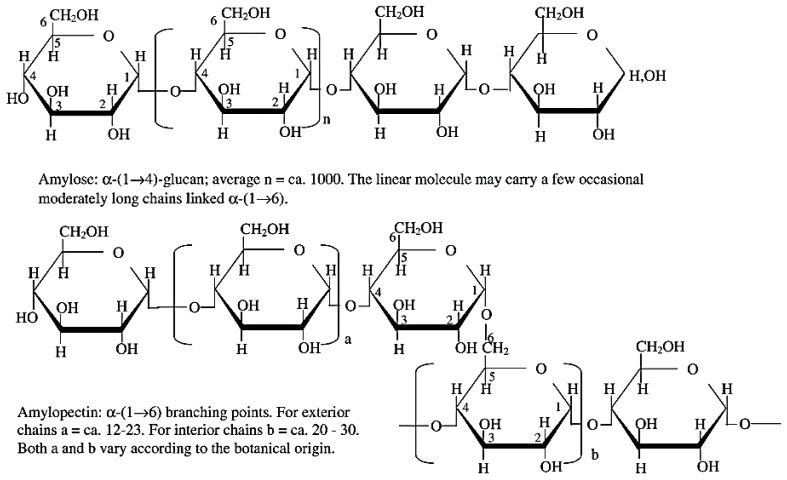
Chemical structure of amylose and amylopectin [66], Reproduced with permission from Elsevier.

Starch is a natural polymer and widely used in several applications including food, paper-making, additives, and adhesives [64,65]. Starch is the admixture of two distinct polysaccharide fractions: amylose and amylopectin; both are composed of glucose with different sizes and shapes [66]. Figure 3 illustrates the general structure of amylose and amylopectin. The ratio of these two components varies based on the botanical origin of the starch. Amylose has a relatively long linear α-glucan structure composing of approximately 99% (1 → 4)-α-linkages and slight (1 → 6)-α-linkages [66,67,68]. The molecular weight of amylose is around 10^5^–10^6^ Da with the degree of polymerization (DP) around 500–5000 [69]. Amylose structure has the branch number around 9–20 per molecule and each chain is built of 200–700 glucose units [66,69]. However, amylopectin is a highly branched polysaccharide which mainly contains 95% (1 → 4)-α- and 5%–6% (1 → 6)-α-linkages [66,67]. The molecular weight of amylopectin varies in the range of 10^7^–10^9^ Da with DP of around 9600–15,900. The molecular chain of amylopectin is made up of 18–25 units, which is shorter than the amylose molecular chain [66]. The morphology of starch is the mixture of loose amorphous regions in highly crystalline regions due to the hydrogen bonds between the starch molecules [70]. The crystalline regions inhibit the penetration of water and chemical components into the structure resulting in a higher gelation temperature and a lower reactivity of the starch. Therefore, it has been recommended to perform some modifications on the crystalline region of starch or to reduce the size of the crystalline segments. Various modification methods have been proposed to decrease the starch crystallinity, including chemical treatments (oxidation [71,72], esterification [65], and cationization [73,74]), physical treatments (mechanical activation [70], microwave [75,76], ultrasonic degradation [77,78], heat-moisture treatment [79]) and enzymatic treatments [80,81,82].

### 3.2. Starch-Based Wood Adhesives

Starch is a promising feedstock for the development of bio-adhesives due to its accessibility, easy process, low cost, good adhesion, and good film formation properties [83,84]. Starch has three hydroxyl groups on C2, C3, and C6 positions in each glucose unit with the ability to form hydrogen bonding. The high affinity of starch to water via hydrogen bonding leads to its poor water resistance and slow drying of starch-based adhesives. The direct use of starch in bio-adhesives is not successful since the adhesive has a poor storage stability and poor bonding strength. Hence, it is necessary to modify the starch to improve its performance for adhesive applications.

Esterification is a typical chemical modification of starch by converting hydroxyl groups into esters to improve the hydrophobicity of starch. The water absorptivity and water solubility of the esterified starch depend on the chain length of the esterification agent and the degree of esterification (DS). A esterified corn starch was obtained by reacting with maleic anhydride and then crosslinking with a polyisocyanate pre-polymer [65]. The addition of polyisocyanate pre-polymer into adhesive improved its dry and wet shear strength and thermal stability. The optimal amount of pre-polymer was found to be at 10 wt %, resulting in the dry and wet shear strengths of 12 and 4 MPa, respectively; they are sufficient to satisfy the Chinese national standard HG/T 2727-2010 requirements. Tan et al. [83] modified a starch-based adhesive by addition of blocked isocyanate and auxiliary agent. The dry and wet bonding strength reached the peaks when the mixing ratio of starch and blocked isocyanate was 100/20 and 100/25, respectively. Furthermore, bonding the isocyanate to the starch-based adhesive could reduce the viscosity of the adhesive. Introducing additive bentonite could thicken the adhesive and improve its water resistance. Two optimal percentages of bentonite added to the adhesive were found at 4% and 6%; and their corresponding wet (and dry) bonding strength became 0.8 MPa (and 1.4 MPa) and 1.56 MPa (and 0.72 MPa), respectively. Wang et al. [85] developed a novel bio-adhesive from grafting of vinyl acetate onto waxy cornstarch, in which VAc reacted with the hydroxyl group of the glucose to generate an ester linkage. Grafting copolymerization of the synthetic polymers onto the starch backbone enhanced the bonding properties of the starch. The best starch/monomer ratio was 1:1.2 (*w*/*w*), yielding an optimal shear strength of 4.3 and 2.17 MPa in the dry and wet state, respectively. Furthermore, in comparison to the blend of a commercial polyvinyl acetate (PVAc) emulsion/gelatinized starch, the grafted starch exhibited an increase in dry and wet shear strengths, and the water resistance by 59.4%, 321%, and 61.1%, respectively. Confocal Raman microscopy (CRM) was applied to evaluate the homogeneity of the grafted vinyl acetate and the quality of the resultant starch-based wood adhesive (HSSWA) [13]. It was found that the ester groups were not evenly bonded among starch granules and the grafted starch showed a higher band area than the blended starch due to the higher grafting level (G). In a similar study, a bio-adhesive was produced based on a wild acorn starch containing 76.3% amylopectin [86], where wild acorn starch was modified by isocyanate, and then mixed with various mass ratios of polyvinyl acetate to formulate the adhesives for plywood application. The bonding strength reached the minimum requirement based on type II Chinese standard (0.8 MPa) when the amount of isocyanate and the polyvinyl acetate emulsion was 15% and more than 40% in the formulated adhesive, respectively.

Starch can also be chemically modified by an oxidation process to form a more reactive wood binder. Yang et al. [87] prepared a cornstarch adhesive through an oxidation-gelatinization process and then characterized its rheological properties. The apparent viscosity was measured as a function of several parameters such as temperature, starch to water ratio, and shear rate. It was found that the apparent viscosity reached a maximum value at 10 °C and then reduced with increasing the temperature; and, the viscosity decreased slightly on increasing the shear rate from 6–60 RPM. The starch adhesive behaved as a pseudo-plastic fluid.

**Figure 4:**
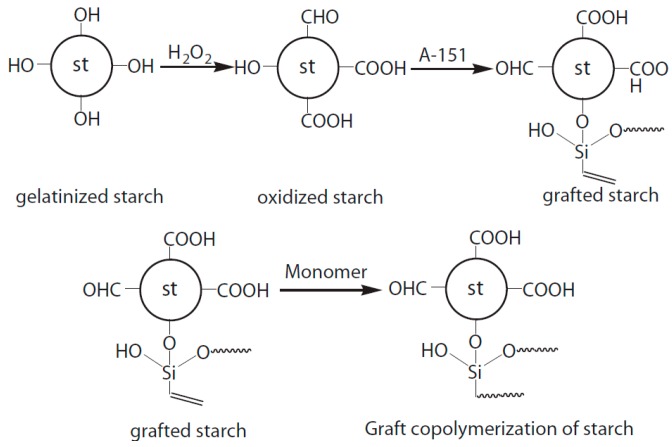
Reaction pathway for the synthesis of starch-based adhesive [88], Reproduced with permission from Elsevier.

An innovative starch-based adhesive was formulated by the addition of a silane coupling agent (CH_2_=CH–Si (OC_2_H_5_)_3_, A-151, as a cross-linking agent), an olefin monomer (butyl acetate and vinyl acetate, as a co-monomer) and hydrogen peroxide (as an oxidant) [88]. Figure 4 shows the reaction pathway for the synthesis of this starch-based adhesive. The hydroxyl groups of starch were converted into carboxyl and aldehyde groups in the presence of the oxidant agent. The graft copolymerization improved the thermal stability, water resistance, and the bonding strength of the prepared adhesive. The optimization process suggested that the addition of 9 wt % of coupling agent and 3 wt % of oxidant agent could produce a modified starch-based adhesive with dry and wet bonding strengths at approximately 7.88 and 4.09 MPa, respectively.

Amini et al. [89] modified corn starch with 25% glutardialdehyde solution (GDA) and then used the modified starch as a binder for the production of rubberwood particleboards in three different densities, i.e., 0.6, 0.7, and 0.8 g/cm^3^. The modulus of rupture (MOR) and the internal bond strength (IB) of all the prepared panels were acceptable. However, the water resistance of panels was required to be improved by the addition of water repellent agents. Xu et al. [64] developed a biodegradable adhesive based on a cassava starch, an olefin monomer (vinyl acetate (VAc)), and butyl acrylate (BA) as a co-monomer. The prepared adhesive showed a low level of total volatile organic compound emissions (TVOC) around 81.2 g/L and a good storage ability at room temperature. The addition of the hydrophobic BA enhanced the bonding strength and the emulsion stability of the starch-based adhesive.

**Figure 5:**
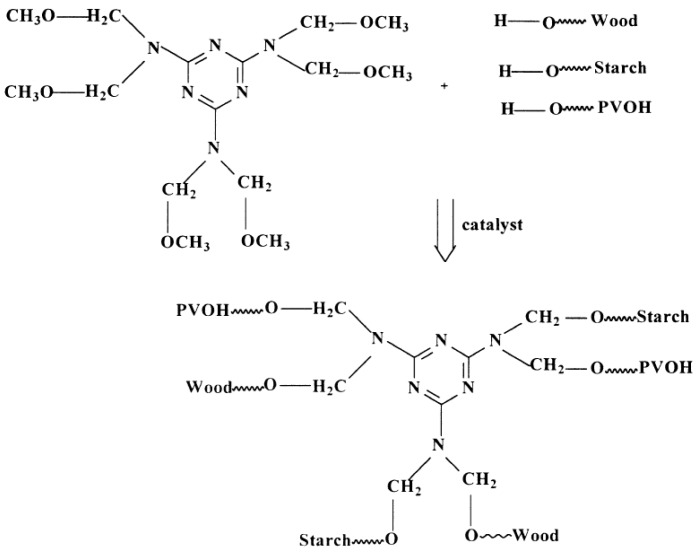
Possible cross-linking reaction of starch-based wood adhesive in the presence of hexamethoxymethylmelamine (Cymel 323) [91], Reproduced with permission from Elsevier.

Wang et al. [90] formulated a low-cost starch-based aqueous polymer isocyanate (API) wood adhesive containing acid thinning, oxidizing corn starch, diphenylmethane diisocyanate (pMDI), styrene butadiene rubber (SBR), and polyvinyl alcohol (PVOH). The optimum formulation with the best bonding strength consisted of 45 g of the modified starch, 18% of pMDI, 10% of PVOH, and 3% of SBR. It was observed that the micro-morphology of starch was altered during the synthesis of API adhesive. This could be associated with the possible participation of starch in chemical reactions with other ingredients in the API adhesive. Another modified starch-based adhesive was produced through a chemical cross-linking with polyvinyl alcohol (PVOH) in the presence of hexamethoxymethylmelamine (Cymel 323) [91]. Cymel 323 could link to other compounds (Starch, PVOH, and wood) through a transetherification reaction as illustrated in Figure 5. The addition of Cymel 323 increased the viscosity of the system with a higher moisture resistance and accelerated the curing process. The adhesive containing 15% of Cymel 323, 7% of latex and 0.9% of citric acid exhibited an optimum performance, which can be cured at 175 °C for 15 min. In addition, no traces of the growth of microorganism and degradation were detected on the surface of adhesives after exposing to 50% RH (rice husk) for a year. It was also found the addition of latex (UCar 443, a copolymer of acrylic and methacrylic acids and their derivatives) to the formulated adhesive improved the moisture resistance and tensile strength of the prepared plywood panels and reduced the solution viscosity [92].

The carboxymethylation process was applied to improve the properties of starch-based wood adhesive. Zhang et al. [93] optimized the synthesis of a carboxymethyl starch-based based on a quadratic orthogonal rotation combination design. Enhancements in water resistance and freeze-thaw stability of adhesive were observed after the carboxymethylation process. In another work, Oil Palm starch was firstly crosslinked by phosphoryl chloride (POCl_3_) and then reacted with sodium chloroacetate (C_2_H_2_ClNaO_2_) to form a carboxymethylated starch—this process was previously reported by Kim and Lim [94]—as a starting material for the production of the starch-based wood adhesive [95]. The modulus of elasticity (MOE) and internal bonding (IB) of the particleboards bonded with the carboxymethylated starch met the requirements of the Japanese Industrial Standards (JIS) except for the modulus of rupture. An addition of 2% of urea formaldehyde to the carboxymethylated starch adhesive could achieve the minimum values specified by the JIS standard.

Moubaril et al. [96] reported a partial substitution of phenol-formaldehyde with cornstarch-quebarcho tannin-based resin in the fabrication of plywood. The optimum replacement was around 20% (containing 15% cornstarch and 5% quebracho tannin). The NMR and FTIR results showed that no reaction occurred between phenol-formaldehyde and the bio-based resins. The addition of cornstarch-quebarcho tannin-based resin improved the water resistance and reduced the formaldehyde emission level of the phenol-formaldehyde resin. They also produced a non-volatile and non-toxic adhesive from the chemical reaction of wattle tannin, cornstarch-based adhesive, and hexamethylenetetramine (hexamine) as a hardener for interior plywood [97]. The plywood bonded with cornstarch-tannin adhesive showed an excellent performance in terms of mechanical properties in comparison with a commercial phenol-formaldehyde adhesive. The optimum curing process was 4 min reaction at 170 °C. The addition of tannin made the starch-based wood adhesive less toxic and more environmentally friendly; it also shortened the reaction time.

Wang et al. [98] investigated the effect of urea on the freeze-thaw (F/T) stability of the starch-based wood adhesive to develop an adhesive with a longer shelf life. The sample went through F/T cycles by freezing under 4 °C for 22 h and thawing under 25 °C for 2 h (repeated up to 10 cycles). The results showed that adding urea into the formulated adhesive improved viscosity, stability, and bonding properties. The improvement could be associated with the ability of urea to retard the retrogradation of the starch molecules via the disruption of hydrogen bonds so as to improve the storage stability. Desai et al. [99] made a polyurethane wood adhesive through a transesterification reaction of potato starch with a vegetable oil such as argemone oil or castor oil. The polyols with high hydroxyl content improved the bonding strength and was comparable with the commercially available wood adhesives. The highest joint strength was achieved at an approximate NCO/OH ratio of 1.3 with a good water resistance in both cold and hot states. However, the formulated adhesive showed medium resistance to acid condition and weak resistance to alkaline condition.

**Figure 6:**
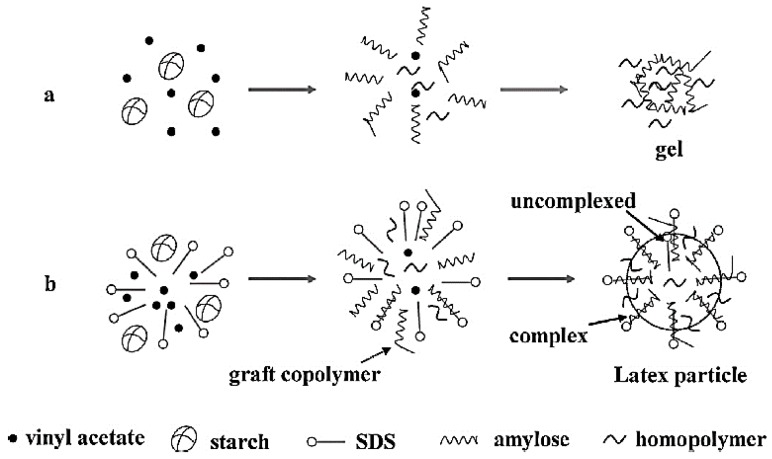
The schematic structure of starch-based wood adhesive (**a**) with sodium dodecyl sulfate (SDS); (**b**) without SDS [101], Reproduced with permission from Elsevier.

Another strategy to improve the performance of starch-based adhesives is to incorporate fillers or additives into the formulated adhesives. Wang et al. [100] showed that the bonding strength, water resistance as well as thermal stability of the starch-based adhesive was improved significantly by silica nanoparticles. An addition of 10% silica led to the best shear strengths of 5.12 and 2.98 MPa in dry and wet states, respectively. The influence of Montmorillonite (MMT) on the performance of a corn starch-based wood adhesive was studied as well [14]. With addition of 5% of Montmorillonite, the shear strength was raised by approximately 2-times and 1.2-times in the dry and wet conditions, respectively. The addition of MMT also affected the shear rheology of the adhesive. For both low and high shear rates, the shear viscosity of the sample with MMT was higher than that of the adhesive without MMT. The sample containing 5 wt % of MMT showed good mobility; its viscosity remained at a low level around 24.4 Pa.s. The thermal property of the starch-based adhesive was improved in the presence of 5 wt % of MMT; thermal degradation occurred at a higher temperature. It appeared that the addition of MMT enhanced the overall performance of starch-based adhesive. The effect of sodium dodecyl sulfate (SDS, an anionic surfactant) on the performance of a starch-based adhesive was evaluated by Li et al. [101]. Figure 6 shows the proposed structure of starch-based adhesive with or without SDS. SDS was added into the synthesis reaction before the graft copolymerization of vinyl acetate onto starch. The structure of the prepared starch-based adhesive was characterized by the blue value (i.e., the color variation from blue to purple) and DSC measurement. A 63.8% reduction in the blue value, and the detection of an endothermic phase transition (Helical transition) by DSC confirmed the formation of the amylose–SDS complexes in starch-based adhesives when the content of SDS is in the range of 1.5%–2% of the dry starch basis. The addition of SDS resulted in a slight drop in shear strength but improved both the mobility and storage stability of the adhesive.

## 4. Plant Protein

Plant protein is another natural resource to produce environmentally friendly wood adhesives. In general, protein-based adhesives have high viscosity, short pot life, and are highly sensitive to water [102], which are the key technical obstacles for their wide applications. It is required to modify the structure of proteins to improve the water resistance, process-ability, and bonding strength of protein-based adhesives. Several studies have been conducted on the manufacture of protein-based adhesives derived from different crops such as soy [103,104,105], canola [102,106], cottonseed [107,108], wheat gluten [109,110], zein [16], and peas [16]. The key findings of these recent reports are summarized in this section based on the types of plant protein.

### 4.1. Soy Protein-Based Wood Adhesives

Soy protein is one typical type of plant protein used to substitute synthetic resins for wood adhesives. The application of soy protein-based adhesives has been limited because of the poor water resistance. To overcome this problem, several methods have been employed to improve the performance of soy protein-based adhesives such as cross-linking [111,112], enzymatic modification [104,113], chemical denaturation [113,114], and the addition of additives [115]. Lei et al. [111] investigated the cross-linking of soy-based adhesives with different types of cross-linkers: epoxy resin, melamine-formaldehyde, and a mixture of epoxy resin and melamine-formaldehyde. The water resistance of soy-based adhesives was improved after modification regardless of the type of cross-linker; the mixture of cross-linkers resulted in the greatest improvement and could be attributed to the chemical reaction of epoxy and melamine-formaldehyde with hydroxyl (–OH) and amine groups (–NH) of soy protein, respectively.

Adding hydrophilic polyols such as ethylene glycol, and diethylene glycol into soy protein-based adhesives improved the wettability and wet adhesion strength by 30% due to the formation of intermolecular hydrogen bonding [103]. However, the addition of polyethylene glycols with a higher molecular weight (greater than 2000) was found to have only a slight improvement in wettability of soy-based adhesive because of a weak interaction with protein matrix. Liu et al. [112] proposed a method to modify soy protein with maleic anhydride. Maleic anhydride was grafted onto protein via amide and ester linkages. The grafted soy protein was combined with polyethylenimine (PEI) to produce an acceptable adhesive. The bonding strength and water resistance of the adhesive were influenced by several factors including the content of maleic anhydride, content of PEI, molecular weight of PEI, and the curing conditions of wood composites. Rassam [114] modified the soy meal through a denaturalization process. The modified soy meal was incorporated in the synthesis of phenol-formaldehyde resin. The resultant adhesive was used as a binder in the recycled old corrugated container (OCC)–wood composites. It was reported that the addition of 10 wt % of soy/PF resin into wood chips containing 25% to 50% OCC could generate wood boards for exterior application. Ciannamea et al. [116] modified both rice husk (RH) as wood chips and soy-protein to enhance the performance of the formed medium-density particleboards. Rice husk was chemically treated with sodium hydroxide or sodium hydroxide and hydrogen peroxide (bleaching process) to eliminate lignin, hemicellulose, and silica from RH. Then, the modified RH were bonded with alkali-treated soy protein adhesive. The morphology of RH was altered in the modification process so as to provide a better mechanical interlocking and more hydrogen bonding between the hydroxyl groups of cellulose and the polar groups of the unfolded soy protein; these changes resulted in greater adhesion strength. Chen et al. [115] characterized the effects of carbohydrates on adhesion strength and water resistance of soy-based adhesives. On increasing the carbohydrate content in soy-based adhesive, the hydrophobicity and degree of cross-linking of the cured soy-based adhesive were reduced. However, increasing the content of sucrose and glucose in the carbohydrate improved the hydrophobicity and bonding strength of the adhesives since a larger content of sucrose and glucose can increase the extent of the Maillard reaction. Khosravi et al. [117] suggested that soy protein could be used as a binder only in a dispersion state and not in powder state. It was found that the dispersion time had a significant effect on the water resistance and mechanical properties of the ultimate product.

### 4.2. Canola Protein-Based Wood Adhesives

**Figure 7:**
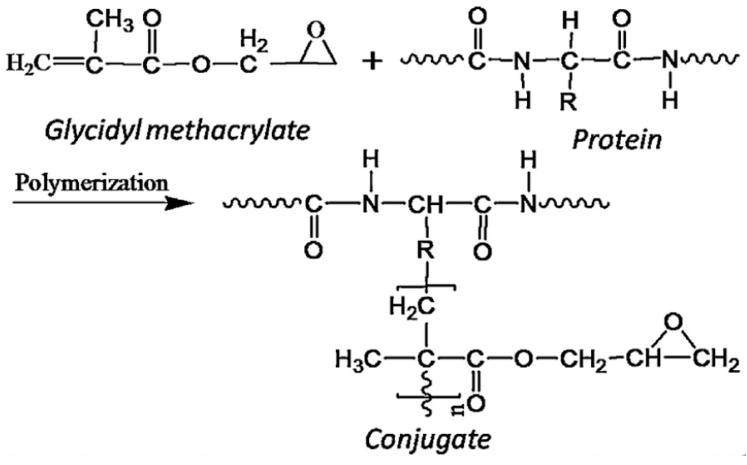
The chemical reaction between canola protein and glycidyl methacrylate [106], Reproduced with permission from Elsevier. R: Possible functional groups (COOH, SH, OH, NH_2_).

Canola is the second largest oilseed products after soy worldwide. Canola has 42–43 wt % oil with a 30–45 wt % protein content in the defatted meal [106]. Canola protein is mainly composed of 60% cruciferin and 20% napin. Napin and cruciferin belong to albumin and globulin protein categories, respectively. Napin is classified as a basic protein (isoelectric point: 11 pH) with the average molecular weight 12–15 kDa and stabilized by disulfide bonds. Cruciferin is a neutral protein with the molecular weight of approximately 300 kDa [106,118]. Canola protein is generally used as animal feed with limited application in other industries. However, some research has shown that canola protein can be converted into valuable materials. Li et al. [102,118] modified canola protein using sodium bisulfite (NaHSO_3_) at different pH values. On increasing the concentration of NaHSO_3_, the recovery of canola protein increased, but the purities decreased. When canola protein was modified by 3 g/L NaHSO_3_, the flow-ability and hand-ability of the adhesive were improved dramatically without compromising its wet shear strength. The physicochemical studies showed that the average molecular weight, thermal transition temperature, and the apparent viscosity of canola protein were reduced after NaHSO_3_ modification. Wang et al. [106] grafted glycidyl methacrylate on canola protein via free radical polymerization to make a wood adhesive. Figure 7 illustrates the chemical reaction between canola protein and glycidyl methacrylate. The conjugating process improved the thermal stability and water resistance of protein-based adhesive via the formation of hydrogen and covalent bonds. The conjugate with 81.97% degree of grafting showed a better bonding capacity due to the mechanical interlocking and chemical reaction with the wood surface.

### 4.3. Cotton Protein-Based Wood Adhesives

Cotton is one of the non-food crops and mainly grown for its fiber. Cottonseed is not consumed directly by humans because it contains toxic gossypol but it can be used to make bio-adhesives. He et al. [107] investigated the adhesive properties of various fractions of cottonseed protein. Two extraction methods were conducted on cottonseed meal in NaCl aqueous solution and phosphate buffer/NaCl solution. The extracted fractions were applied onto maple veneer strips and examined by lap shear tests. The bonding strengths of the extracted fractions were 1.32–1.62 MPa, which were comparable to or even better than the bonding strength (1.32–1.49 MPa) of the original cottonseed. Note that the reported values of glandless cottonseed fractions were smaller than those of the original cottonseed. The soaking experiments showed that no delamination occurred in the wood specimens bonded with water- and buffer-washed adhesives while the original cottonseed meal adhesive-bonded specimens had 20%–30% delamination. It means that the extracted fractions of cottonseed had a higher water resistance compared to the original cottonseed. He et al. [108] also characterized the effect of tung oil on bonding strength and water resistance of cottonseed protein adhesive on maple veneer. The addition of 0.05%–1% tung oil into cottonseed protein adhesive improved the shear strength and water resistance of the bonded wood by 21.1% and 41.3%, respectively. The enhancement in properties could be associated with the ability of tung oil to prevent the adhesive from delamination when exposed to water. In addition, it was found that the hot-pressing temperature had no influence on the water resistance of protein-based adhesives containing tung oil.

Water resistance of cottonseed protein adhesive could be improved by some modifiers and additives as well. For instance, Cheng et al. [119] studied the influence of some additives on the performance of cottonseed- and soy protein-based adhesives and also compared the bonding strength of these two types of protein-based adhesives on different wood veneers. A wide range of additives including amino acids, fatty acids, and small molecules with anionic and cationic charges were used in this investigation. The results indicated that the greatest performance of cottonseed protein adhesive (higher adhesive strength and water resistance) was achieved in the presence of small molecules with anionic charge such as aspartic acid, glutamine acid, acetic acid, adipic acid, and butyric acid. In contrast, no enhancement was detected in the bonding strength of soy protein-based adhesives. Moreover, the bonding of cottonseed protein-based adhesive on various wood veneers including pine, walnut, cherry, and maple was much stronger than soy protein-based adhesive on the same veneer. Cheng et al. [120] investigated the impact of various polysaccharide fillers (xylen, starch, and cellulose) on the performance of soy- and cottonseed-protein adhesive. The bonding strength of protein-based adhesives generally remained unchanged in the presence of polysaccharide up to 75 wt % regardless of the types of protein-based adhesive. However, the effect of polysaccharide filler on water resistance depended on the types of the protein. Soy protein adhesives exhibited lower water resistance in the presence of polysaccharide filler; the water resistance of cottonseed protein adhesives remained unchanged up to 50 wt % of polysaccharide in the ultimate formulations.

### 4.4. Wheat Gluten-Based Wood Adhesives

Wheat gluten (WG) is a by-product from wheat starch processing. Wheat gluten is a complex mixture comprising 80% wheat protein, and the rest is lipids, polysaccharides, and minerals. The wheat protein is equally made of two protein fractions: gliadins and glutenins [15]. Gluten wheat has a high amount of hydrophobic amino acids with isoelectric point around 7 [121]. The average molecular weight of gliadins varies from 30,000 to 60,000 g/mol, and the molar mass of glutenins is in a wide range of 500,000–10^7^ g/mol [15]. The elastic properties of wheat gluten are associated with the glutenin fractions, while gliadins contribute to the viscous properties [15,121,122]. Some studies have been reported on the development of wheat gluten-based wood adhesives. El-Wakil et al. [123] succeeded in modifying wheat gluten in the presence of NaOH and urea and then used a partial substitution of urea-formaldehyde up to 80 wt % with promising mechanical performance. The addition of the modified wheat gluten also improved the thermal stability of the bonded reed particleboard. Lagel et al. [124] incorporated the hydrolyzed wheat gluten in the synthesis of phenol-formaldehyde resin up to 30% while retaining the adhesion properties of the wood joint. Furthermore, the presence of triacetin in the formulation of phenol-wheat-formaldehyde improved the bonding properties.

In another approach, hydrolyzed gluten reacted with glyoxal or formaldehyde and then mixed with tannin/hexamine resin or pMDI to form an adhesive with approximately 90–95 wt % and 70–80 wt % of renewable components, respectively. Both formulations showed acceptable bonding strength for interior grade particleboards. Nordqvist et al. [125] proposed two modification processes: enzymatic hydrolysis and heat treatment, to improve the bonding properties of wheat gluten-based adhesive. When the hydrolysis process was conducted at low levels (0.3%–0.6%) or thermal treatment was performed at 90 °C, enhancements were obtained in water resistance and bonding strength of the bonded beech wood specimens. D’Amico et al. [110] investigated the impact of alkaline and enzymatic hydrolysis treatments on molecular weight distribution of wheat gluten. The alkaline hydrolysis for 4 h had a significant influence on molecular weight of wheat gluten and generated protein fragments predominantly with a molar mass below 30 kDa. While less decomposition occurred during the enzymatic treatment, the main fragments were between 60 and 80 kDa, similar to the native wheat gluten, with no detection of fractions over 100 kDa. Khosravi et al. [121] studied the influences of application process and the dispersion concentration of wheat gluten-based adhesive on the board properties by employing a factorial screening design. The internal bonding results revealed that the 2-step gluing method (i.e., the two separate steps of (1) adding adhesive dispersion and (2) drying the glued chips between each addition), was superior to the one-step gluing process. It is suggested that the 2-step application method prevented the over-penetration of adhesive into wood chips and provided a better bonding strength. The preferable concentration of adhesive dispersion depended on the application method. A less concentrated dispersion was more suitable for the 2-step gluing method, while a more concentrated dispersion showed better performance for the 1-step application process. Nordqvist et al. [15] evaluated the performance of glutenin and gliadin dispersions in sodium hydroxide solution as a denaturing and dispersing agent and compared them with wheat gluten adhesive. The results demonstrated that the tensile shear strengths and water resistance of glutenin and wheat gluten were comparable, while the gliadin dispersion had inferior properties and was significantly affected by the application process. The inferior properties of gliadin could be associated with the extensive penetration of gliadin into the wood fibers; there was not sufficient glue on the surface of the wood to bond them together. In another study, Nordqvist et al. [109] evaluated the bonding strength of soy protein and wheat gluten adhesives in both acidic and alkaline conditions at micro- and nanoscale. In comparison with wheat gluten adhesive, soy protein adhesive showed better shear bonding strength and is appropriate for durability classes D1–D3. Optical microscopy and atomic force microscopy were used to examine the penetration of bio-based adhesives into wood. The results indicated that soy protein adhesive was able to make an appropriate bonding with a lower content compared with the wheat gluten adhesive.

## 5. Evaluation of Wood Adhesives

Based on the above discussion, the performance of three types of bio-based wood adhesives derived from lignin, starch, and plant protein are compared and their advantages and disadvantages are summarized in Table 1. For any type of bio-based adhesives, it is important to effectively evaluate their prospects for wood composite application and understand the adhesive/wood interaction to gain scientific insights to guide the adhesive development in terms of the mechanical strength, water resistance, thermal and rheological properties of the adhesives, as well as the adhesive penetration. In this section, the common mechanical tests of the bonded wood composite, water resistance, and the important adhesive penetration behavior in the wood and the techniques available for characterizing the adhesive penetration are discussed.

### 5.1. Wood Adhesive Bonding Strength Evaluation

**Figure 8:**
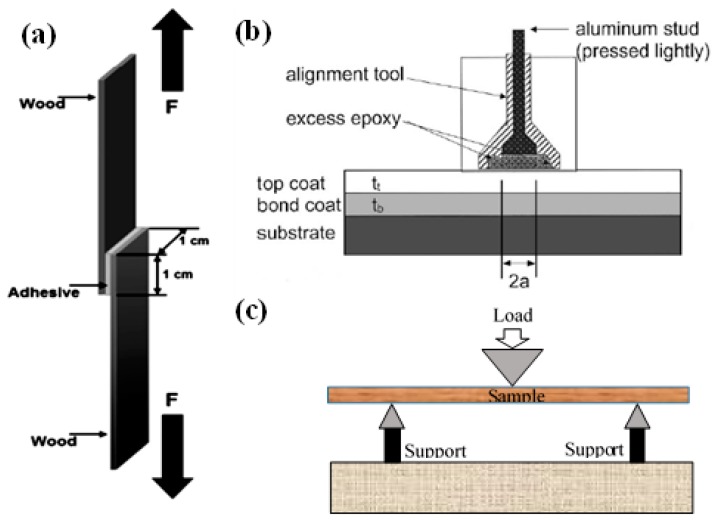
Schematic of wood adhesive (**a**) shear strength test [137], Reproduced with permission from Elsevier; (**b**) pull-off strength test [130], Reproduced with permission from Elsevier; and (**c**) three-point bending strength test.

Shear strength [126,127,128,129], pull-off strength [130,131,132], and bending strength [133] are mainly used to evaluate the bonding strength of wood adhesives and the mechanical properties of wood composites. There are several standards to measure the shear strength of the adhesive bonded wood composites such as the ASTM (Standard Test Method for Strength Properties of Adhesive Bonds in Shear by Compression Loading) D 905, D 4262 and D 2559 [126,131,134,135,136,137], the European Standard EN 205 [97,127,138,139], and the China National Standard (GB/T 17657-2013) [140]. As described in these standards and illustrated in Figure 8a [137]: in the shear test, one end of each wood chip is glued by adhesives and the other end is inserted into the clamps of the tensile machine and moved up at a certain speed until the bonded wood chips are detached. According to the ASTM-D 4541 (1995) pull-off test standard [126], Figure 8b illustrates the setup of the pull-off test. A thin layer of wood adhesive is coated onto the wood surface and the larger head of the aluminum stud is pressed and attached onto the wood adhesive. The tail of the stud is then inserted into the clamp and moved up slowly with a constant speed until the stud detaches from the adhesive/wood surface; the maximum force is the tensile strength of the aluminum stud separated from the wood surface. The bonding strength is calculated through the Equation (1) [134]:

(1) $$\sigma_{\mathrm{bonding}-\mathrm{stength}}=\frac{F_{\max}}{S}$$

in which σ_bonding-strength_ is the bonding strength, *F*_max_ is the maximum force, and *S* is the contact area.

According to the standard ISO 3133 (1975) [133], the bending strength of wood composites is tested by three-point test method in a standard tensile-pressing machine (Figure 8c). The bending strength can be calculated by Equation (2):

(2) $$\sigma_{b}=\frac{3F_{\max}l}{2bh^{2}}$$

In which σ_b_ is the bending strength (*MPa*), *F*_max_ is the breaking force of the sample, *l* is the space between each supporting pin (mm), *b* is the width of the sample (mm), and h is the thickness of the sample (mm). In this method, wood composite is placed onto two supporting points after that the probe is brought into contact with the wood chip; the preload increases slowly at a constant rate until the wood composite breaks. The maximum force *F*_max_ is recorded and used to calculate the bonding strength of the wood adhesive.

To interpret the measured bonding strength of wood adhesives, it is essential to know where the failure or separation occurred in the mechanical tests. There are four main types of failure modes for the adhesive bonded wood composites: (a) cohesive failure of the adhesive; (b) adhesive failure at the interface; (c) mixed failure—a combination of (a) and (b)—and (d) wood cohesive failure or wood failure [99,141,142,143,144]. In cohesive failure of the adhesive, the failure is observed in the adhesive layers. In adhesive failure, the adhesive was detached from the wood at the interface. In the mixed failure mode, both the cohesive failure of the adhesive and adhesive failures at the interfaces are observed. In wood cohesive failure, an entire layer of wood fibers is pulled off from the wood substrate and thus failure occurs in the wood. The percent material failure is often used to describe failure of wood composites, indicating how much of the adhesive or wood substrate is pulled off from the whole contact area under a certain condition. In the proposed four types of failure mode, the cohesion failure of adhesive indicates the weak bonding which is not favored by the industry. The adhesive failure between adhesive and wood implies the better bonding performances of wood adhesives. The mixed failure mode includes the cohesive failure of adhesive and the adhesive failure showing the better interactions between adhesive and wood substrate resulting in a stronger bonding. When the adhesive penetrates into the wood substrate deep enough so as to form mechanical interlocks and other interactions with the wood, a layer of wood would be taken off from the bonded surface. Thus the mixed mode and wood failure are preferred in wood fabrication to obtain well-bonded and high performance wood composites [99,141,142,143,144].

Water resistance of wood adhesive bonding plays an important role in the mechanical properties and the ultimate application of wood composites. The bonded wood composites with high water resistance are suitable for exterior-grade panels, while the bonded panels with mild water resistance are appropriate for interior-grade application. Water resistance can be determined based on ASTM Standard Methods D1183-96 [118], D1151-00 [102,119] and US Voluntary Product Standard PS 1-95 [59]. As described in these standards, the bonded wood panel can undergo various conditions of wet test (WT), water-soaking-and-drying (WSAD) test, and boiling water test (BWT). For the WT, the bonded specimen is soaked in water at 23 °C for 48 h, then evaluated immediately for wet shear strength. For the WSAD test, the bonded panel is soaked in tap water at room temperature for 48 h, then dried at 23 °C for 7 days before testing. For the BWT, the specimen is boiled in water for 4 h, dried for 20 h at 63 ± 3 °C, boiled in water again for 4 h, and then cooled down with water before evaluation. Afterward, the bonding strengths of wood composites before and after exposure to water are compared to determine the water resistance based on the reduction in bonding strength. In a humid environment, the bonding strength of the bonded panel will decrease due to penetration of water molecules into the wood and acting as a plasticizer [100].

### 5.2. Wood Adhesive Penetration

**Figure 9:**
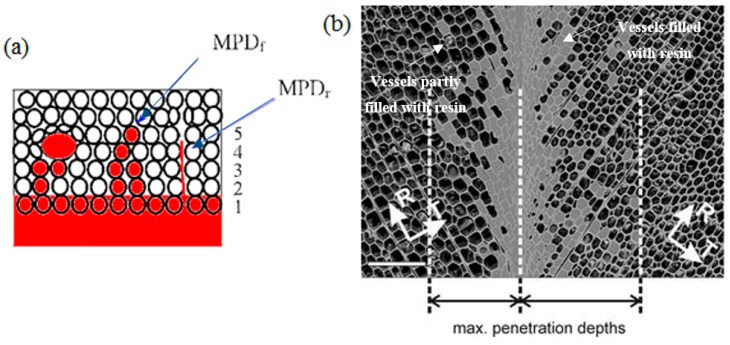
(**a**) Illustration of numbers of filled fibers (FFN), numbers of filled vessels (FVN), maximum penetration depth in fibers (MPD_f_), and maximum penetration depth in rays (MPD_r_) [157], Reproduced with permission from Springer; (**b**) states of adhesives filled in vessels [149], Reproduced with permission from Springer.

A wide variety of wood adhesives have been developed and extensively utilized in industrial processes to manufacture advanced structural wood-based products [138,145,146,147,148]. The main function of wood adhesives is to bond wood panels and small wood pieces together, and form wood composites with the desired dimensions, mechanical properties, and without the natural wood anisotropy [145,149,150]. The bonding interactions between the adhesive and wood are principal factors affecting the performance of wood composites [100,134,151,152]. Therefore, it is essential to characterize the interactions of wood and adhesive to reveal the bonding mechanism [153]. Wood surface has high porosity, high surface energy, and good wettability [145,154,155,156]. Thus, polar liquids are able to wet the wood surface and flow into the internal structures (e.g., vessels and lumens) of wood [145,154]. This phenomenon is defined as adhesive penetration, which has a profound influence on bonding interactions and bonding strengths. As illustrated in Figure 9a, the adhesive penetration has been quantitatively described by four parameters: numbers of filled fibers (FFN, circles filled with red color), numbers of filled vessels (FVN, the whole ray fibers were filled with adhesive, parallel to the bond line which is 1), maximum penetration depth in fibers (MPD_f_, the furthest circle filled with red in perpendicular direction to the bond line which is 5), and maximum penetration depth in rays (MPD_r_, parallel direction to the bond line, there four rays of circles are filled with red color) [157]. In most cases, the adhesive penetration in wood is not uniform and involves different depths e.g., fully filled vessels, partially filled vessels, and interactions on wood cells [155] (Figure 9b). The motion of adhesives in the longitudinal direction of wood is more significant than in the lateral direction due to the particular arrangements of vessels/lumens in the longitudinal direction (connecting end-to-end) [145]. Penetration depth has a close relationship with the ultimate performance of wood composites. An excessive penetration causes an increase in manufacturing cost and defects on the bond line. In contrast, insufficient penetration leads to a reduction in bonding strength due to limited contact areas for formation of chemical/mechanical bonding [157,158,159].

Adhesive penetration can be divided into two categories: (1) gross penetration at the microscale [155] and (2) cell-wall penetration at the nanoscale [160]. In gross penetration, the liquid adhesives wet and spread on the wood fibers, flowing into micro-sized pores and fully or partially filling the lumens, vessels and encapsulating fractures [145,155]. There are two driving forces for gross penetration: hydrodynamic flow and capillary action [145,159]. In cell-wall penetration, liquid adhesives diffuse from outside to cell walls and micro-cracks, swelling and plasticizing the cell wall [145,159]. In addition to the penetration phenomena, the formation of adhesive bonds has been described by various interaction mechanisms such as mechanical interlock, chemical covalent bonding, electrostatic induced interaction, wetting induced interaction, diffusion induced interaction and van der Waals or hydrogen bonding interactions [152]. Among those interactions, mechanical interlock is the primary interaction between wood and adhesive which controls the properties of bond line and affects the performance of wood composites [152].

#### 5.2.1. Factors Affecting Wood Adhesive Penetration

Adhesives penetration is affected by wood property (e.g., soft-/hardwood [159], surface roughness [151], pore size [161], growth ring orientation [162], and wettability [139]), adhesive property (e.g., molecular weight [158,163], viscosity, and solid fillers effect), and processing conditions (temperature, pressure, and moisture). Paris et al. [159] investigated the influence of two types of wood, i.e., softwood (Douglas-fir) and hardwood (hybrid polar), on adhesive penetration. It was found that the penetration of adhesive is much faster in softwood than in hardwood. The fast penetration in softwood could be associated with its highly ordered and well-aligned tracheid, as well as its relatively uniform pore size. In contrast, hardwood has randomly distributed vessels with a wide size distribution, resulting in a random penetration of adhesives [164]. Surface roughness also affects the bonding performance since it is directly related to the contact area between adhesive and wood. Follrich et al. [151] studied the effect of five wood fibers with different roughness on the bonding strength. The results illustrated that the highest tensile strength was achieved for the wood fibers-based sample with the greater surface roughness. Wang and Yan [161] used two types of wood fibers (birch and aspen) to evaluate the effect of pore size on the bonding with phenol formaldehyde resin without any application pressure. The results showed that PF resin readily penetrated into pores larger than 40 µm, partially penetrated into pores ranging from 1 to 40 µm, and barely penetrated into pores smaller than 1 µm.

The nature of the growth orientation of wood also influences the adhesive penetration as well as the bonding strength as reported by Follrich et al. [162], who showed that shear strength decreased with increasing grain angle. Surface wettability of wood can control the flow and motion of liquid adhesives on the wood surface and lead to different degrees of penetration. Wei et al. [139] changed the wettability of birch by using different dyes. It was found that a deeper penetration occurred on a sample with a better wettability (or a smaller water contact angle). Although wood surface and structures have considerable effects on adhesive penetration, factors related to the adhesive properties should also be considered, including the adhesive viscosity, molecular weight, and fillers. Johnson and Kamke [158] demonstrated that the adhesive with a larger molecular weight (≥10,000 g/mol) had a limited penetration while the adhesive with a smaller molecular weight (≤10,000 g/mol) had a significant penetration into wood fibers. In another study, Laborie et al. [163] showed that a lower molecular weight PF had better penetration than a high molecular weight PF. It could be related to the higher mobility of low molecular weight PF and the formation of better nanoscale interactions. Cellulose nanofibril (CNF) was used as a filler in urea-formaldehyde (UF) adhesive; the added CNF increased the viscosity of UF resulting in a thicker bond line and a smaller penetration [165]. Additionally, the application processes have apparent effects on adhesive penetration. For example, a proper processing pressure is required to form a desired penetration. Cheng and Sun [166] found that either high or low pressure was not good for penetration. Extra high pressure might destroy the interlayers leading to a poor penetration while at low pressure, there is insufficient contact between the adhesives and wood resulting in a poor penetration.

To predict the penetration of liquid adhesives in the porous wood, Darcy’s law has been used to describe the flow of liquid adhesive into wood fiber under ideal conditions [167]:

(3) $$Q=K\cdot \frac{A}{L}\cdot \frac{1}{\eta }\Delta P$$

In which, *Q*, *K*, *A*, *L*, η, and ∆*P* are the liquid volume flow rate (m^3^·s^−1^), specific permeability of wood (m^2^), area perpendicular to the liquid flow (m^2^), sample length in the direction of flow (m), dynamic viscosity of the liquid (Pa·s) and the pressure drop (Pa), respectively. This equation illustrates the adhesive penetration is determined by the factors of the processing pressure ∆*P*, liquid viscosity η and the wood permeability to liquid *K* supporting the characterization of wood adhesive penetration at the theoretical level. Furthermore, the distribution of adhesives in wood was evaluated by using a non-destructive transmission visualization technique and described by the Lambert-Beer Law [167]:

(4) $$I=I_{0}e^{-\mu d}$$

(5) μ= μ ′ρ

In which, *I*, *I_0_*, μ, μ’, and *d* are the radiation intensity that passes through the bonded wood (cm^−2^·s^−1^·A^−1^), the radiation intensity before adhesive penetration (cm^−2^·s^−1^·A^−1^), the attenuation coefficient (cm^−1^), mass density of liquid adhesive (kg·m^−3^), mass attenuation coefficient of wood (cm^2^·g^−1^) and penetration depth (cm). These equations highlight that the adhesive penetration is the result of the synergetic effects of multiple factors such as the intrinsic property of wood and the property of adhesive.

#### 5.2.2. Characterization Techniques of Wood Adhesive Penetration

The penetration of adhesives into wood fibers likely causes changes in the wood properties such as the morphology of interlayer, elastic modulus difference, composition changes, polymerization etc. [145,149,150,153,160,163,168,169,170]. Both quantitative and qualitative techniques have been developed to observe adhesive penetration at the micro- and nanoscales. These techniques include optical microscopy techniques [145], electron microscopy techniques [145], spectroscopy techniques [171], and tomography techniques [172]. There are many optical microscopy techniques such as fluorescent microscopy [149,158,168], scanning thermal microscopy (SThM) [160], and confocal laser scanning microscopy (CLSM) [170,173] that have been used for examining adhesive penetration. For the electron microscopy method, the widely used methods include scanning electron microscopy (SEM) [174], transmission electron microscopy (TEM) [154], energy-dispersive X-ray analysis (EDAX) [175], electron energy loss spectroscopy (EELS) [175], and X-ray tomographic microscopy (SRXTM) [135]. The common spectroscopy techniques to characterize adhesive penetration are infrared spectroscopy (FTIR) [171], nuclear magnetic resonance spectroscopy (NMR) [176], Raman spectroscopy [177], X-ray microtomography (XMT) [168], and micro X-ray computed tomography (XmCT) [172] which are all considered as tomography techniques. The comparison of these techniques is listed in Table 2 in terms of their applications, advantages, and disadvantages.

The penetration of conventional wood adhesives into wood chip has been widely studied. However, there is no published work on penetration of bio-adhesives into wood fiber. As mentioned above, the adhesive penetration has a significant influence on bonding interactions and bonding strengths of wood composites. So, it is necessary to conduct more studies on the penetration of bio-adhesive to determine the optimum adhesive properties and the best preparation conditions to reach the bio-based wood composite with the greatest performance.

## 6. Conclusions and Perspective

This paper reviews the recent development of biopolymers including lignin, starch, and plant proteins as promising candidates in the synthesis of wood adhesives. In most studies, the replacement of petroleum-based resins by native lignin has been limited to a low level of substitution. After a chemical modification process of lignin, the level of substitution increased (in some cases up to 50 wt %) with acceptable physical and mechanical properties. On the other hand, the petroleum-based adhesives can be fully replaced by starch- or plant protein-based adhesives, but water resistance and bonding strength of the ultimate products are lower. The pretreatment of starch and plant proteins or the incorporation of some additives has been used to improve the water resistance and bonding strength of the bonded wood panels. These results suggested that more petroleum-based adhesive could be replaced by the bio-based adhesives with similar performances in the near future. In addition, it was found that the adhesion bonding strength is significantly affected by the penetration of adhesives into wood fiber. Therefore, the mechanism of penetration, the factors affecting the penetration, and various techniques for measuring the penetration should be examined in parallel to the global mechanical strength evaluations.

We expect that increasing amounts of bio-based (lignin, starch, proteins, etc.) adhesives will be used in the construction of wood composites to reduce negative environmental impact of current wood adhesives and to meet the societal need of developing sustainable materials and economy. However, a complete replacement of petroleum-based wood adhesives with bio-based adhesives will be unlikely in the near future because of the relative poor water resistance and bonding strength of the bio-based adhesives. To broaden the applications of bio-adhesives, it is necessary to chemically modify bio-based polymers to make them more compatible with the current synthetic wood adhesives and enhance their bonding strength and water resistance. Research and technological development of bio-based polymers that can be solely used as wood adhesives is much needed in the field. In addition, the adhesive bonding properties of the bio-based adhesives on wood are still less investigated in comparison to those of the petroleum-based synthetic adhesives; in particular, the adhesive penetration and adhesion in the wood surface and internal structures and the relationship to the overall bonding strength and reliability should be investigated to determine the effective factors for formulating bio-based adhesives with optimum performance.

## Figures and Tables

**Table 1:** Advantages and disadvantages of bio (lignin, starch and plant protein)-based wood adhesives.

Type of Bio-Adhesive	Advantages	Disadvantages
Lignin-based wood adhesive	Utilization of by-products from paper pulping industries Improving the thermal properties Improving the modulus of elasticity Improving the water resistance Good bonding strength	Low level of substitution (max lignin content ≈ 50 wt %) Decreasing the curing rate Increasing the viscosity of adhesive Need chemical modification to improve its reactivity Solubility depends on the type of lignin
Starch-based wood adhesive	High level of substitution Good bonding strength Good film formation property	Poor water resistance Slow drying process Poor storage stability Need pre-treatment to improve the water resistance
Plant protein-based wood adhesive	High level of substitution Good adhesion strength Improving thermal stability	Need pre-treatment to improve the water resistance Poor water resistance

**Table 2:** The comparison of common techniques used in adhesive penetration characterization.

Technique	Application	Advantages	Disadvantages	Reference
Scanning probe microscopy (SPM)/nanoindentation	Cell-wall penetration	Adhesives penetration map	Modulus difference required, two techniques combination	[136,164,178]
Transmitted microscopy	Gross-penetration	Rapid, quantitative evaluation, color contrast	Specimen preparation difficult	
Fluorescent microscopy	Gross-penetration	High color contrast, color filtering, rapid, quantitative measurement	Fluorescer stain	[136,155,158,164,172]
Fluorescent infrared spectroscopy (FTIR)	Gross-penetration	Chemical bonding	No penetration depth and bond line information	[171]
Confocal laser scanning microscopy (CLSM)	Gross-penetration Cell-wall penetration	Adhesive distribution and penetration, 3D view, penetration to single fiber	Low scanning speed, low resolution in Z-direction, image damage	[170,173]
X-ray photoelectron spectroscopy (XPS)	Cell-wall penetration	Penetration to single fiber, quantification measurement	No penetration depth and bond line information, limitation in large scale	[148,169]
Scanning electron microscopy (SEM)/energy-dispersive X-ray analysis (EDAX)	Gross-penetration Cell-wall penetration	Adhesive distribution, penetration, bond line morphology	Gray image, poor contrast, quantitative measurement difficult, large excitation volume	[150,174]
Transmission electron microscopy (TEM)	Cell-wall penetration	Adhesive penetration, bond line morphology, morphology of diffusion in cell wall	Gray image, poor contrast, quantitative measurement difficult, slow	[174]
Scanning thermal microscopy (SThM)	Cell-wall penetration	Distribution at bond line area, high spatial resolution, simple specimen preparation, specimen preparation is simple	Rely on thermal conductivity difference, assisted by AFM, resolution depend on surface height variation, smaller image size	[149,160]
Electron energy loss spectroscopy (EELS)	Gross-penetration Cell-wall penetration	Monitoring penetration, high resolution, adhesive distribution	Combine with transmission electron microscopy, expansive, slow, radiation damage	[161,175]
13C CP/MAS NMR	Gross-penetration Cell-wall penetration	Nanoscale observation, cell-wall penetration, relationship of molecular weight and penetration	Lack of morphology analysis, distribution and penetration depth	[152,171,176]
X-ray microtomography (XMT)	Gross-penetration Cell-wall penetration	3D view, pattern of adhesive	Gray image, poor contrast	[168]
Micro X-ray computed tomography (XmCT)	Gross-penetration Cell-wall penetration	Adhesive distribution, penetration, high resolution, 3D view	Size limitation, pre-treatment of adhesives	[135,172,179]
Raman spectroscopy	Cell-wall penetration	Higher lateral resolution, interlayer composition measurement, adhesive diffusion detection	Not suitable for bond line morphology characterization, penetration depth and adhesive distribution	[177,180]
